# Expression of *GOT2* Is Epigenetically Regulated by DNA Methylation and Correlates with Immune Infiltrates in Clear-Cell Renal Cell Carcinoma

**DOI:** 10.3390/cimb44060169

**Published:** 2022-05-25

**Authors:** Wallax Augusto Silva Ferreira, Edivaldo Herculano Correa de Oliveira

**Affiliations:** 1 Laboratory of Cytogenomics and Environmental Mutagenesis, Environment Section (SAMAM), Evandro Chagas Institute (IEC), BR 316, KM 7, s/n, Levilândia, Ananindeua 67030-000, PA, Brazil; ehco@ufpa.br; 2 Faculty of Natural Sciences, Institute of Exact and Natural Sciences, Federal University of Pará (UFPA), Rua Augusto Correa, 01, Belém 66075-990, PA, Brazil

**Keywords:** KIRC, *GOT2*, multi-omics, epigenetics, immune cell infiltration

## Abstract

Clear cell renal cell carcinoma (KIRC) is the most common and highly malignant pathological type of kidney cancer, characterized by a profound metabolism dysregulation. As part of aspartate biosynthesis, mitochondrial *GOT2* (glutamic-oxaloacetic transaminase 2) is essential for regulating cellular energy production and biosynthesis, linking multiple pathways. Nevertheless, the expression profile and prognostic significance of *GOT2* in KIRC remain unclear. This study comprehensively analyzed the transcriptional levels, epigenetic regulation, correlation with immune infiltration, and prognosis of *GOT2* in KIRC using rigorous bioinformatics analysis. We discovered that the expression levels of both mRNA and protein of *GOT2* were remarkably decreased in KIRC tissues in comparison with normal tissues and were also significantly related to the clinical features and prognosis of KIRC. Remarkably, low *GOT2* expression was positively associated with poorer overall survival (OS) and disease-free survival (DFS). Further analysis revealed that *GOT2* downregulation is driven by DNA methylation in the promoter-related CpG islands. Finally, we also shed light on the influence of *GOT2* expression in immune cell infiltration, suggesting that *GOT2* may be a potential prognostic marker and therapeutic target for KIRC patients.

## 1. Introduction

Renal cell carcinoma (RCC) is one of the most common malignancies of the genitourinary system, representing 3% of all malignancies in adults [1]. In the subtypes of kidney cancer, kidney renal clear cell carcinoma (KIRC) accounts for about 75% of all RCC [2,3,4]. Indeed, due to the evident complexity from both morphological and molecular points of view [5,6,7,8,9], KIRC patients have heterogeneous clinical outcomes [10,11]. Most KIRC tumors are radiotherapy and chemotherapy-resistant, and ~30% of patients eventually develop metastases [3,12,13]. Thus, it is critical to identify new sensitive tumor biomarkers to advance the prognosis of KIRC.

Current shreds of evidence have demonstrated that KIRC is a metabolic disease [13,14,15,16,17,18]. This metabolic reprogramming is mainly related to loss-of-function mutation (or, less commonly, hypermethylation) in the von Hippel–Lindau (*VHL*) gene. VLH inactivation results in constitutive activation of hypoxia-inducible factors (HIF-1α and HIF-2α), thereby altering many genes involved in angiogenesis, metabolism, chromatin remodeling, extracellular matrix (ECM) and DNA repair [9,15,19,20,21]. Accordingly, mutations in several other genes (e.g., *PBRM1*, *SETD2*, *BAP1*, *KDM5C*, and *MTOR*) have been continuously identified to contribute to the pathogenesis (used to classify tumors into subgroups) and metabolic remodeling process of KIRC [22,23].

The metabolic shift in KIRC tumors covers different pathways and specific intermediates (e.g., amino acids, aerobic glycolysis, and fatty acid metabolism) [16,24], allowing cancer cells to rapidly proliferate, and survive during nutrient depletion and hypoxia, and evade the immune system [14]. Supporting this notion, aberrant tumor growth is promoted by an enhanced supply of specific metabolites, and some of them, such as aspartate (Asp), are limiting in some tumors [25,26,27]. Asp is usually synthesized in the mitochondrial matrix through the sequential actions of *MDH2* and glutamic-oxaloacetic transaminase 2 (*GOT2*) and then transported to the cytosol for use by *GOT1* and other enzymes [28].

*GOT2*, situated on chromosome 16q21, is a crucial enzyme for cancer cell metabolism, (i) mediating the reversible interconversion of oxaloacetate and glutamate into aspartate and α-ketoglutarate, providing energy for tumor cells (Krebs cycle) [29]; (ii) being a key transfer enzyme in the malate-aspartate NADH shuttle activity and oxidative protection [30], maintaining glycolysis, and (iii) participating in the amino acid metabolism of tumor cells [31]. Increasing evidence has shown that dysregulation of *GOT2* expression significantly influences tumor growth and the prognosis of several human neoplasms [30,32,33,34,35,36,37,38]. However, the role of *GOT2* in the development and prognosis of KIRC has not been reported. To address these issues, this study aims to evaluate the expression levels of *GOT2* in KIRC and determine its epigenetic modulation, prognostic value, and correlation with tumor-infiltrating immune cells in KIRC patients through multiple databases.

## 2. Materials and Methods

### 2.1. Differential Expression of GOT2 mRNA and Protein

Initially, pan-cancer analysis of *GOT2* transcription levels was performed via the TIMER2.0 database (Tumor Immune Estimation Resource, http://timer.cistrome.org/, accessed on 1 December 2021) [39], using the differential expression module across all TCGA tumors. The statistical significance computed by the Wilcoxon test was annotated by the number of stars (* *p*-value < 0.05; ** *p*-value < 0.01; *** *p*-value < 0.001).

To keep the focus of our analyses on KIRC tumors, the GEPIA2 database (Gene Expression Profiling Interactive Analysis 2) (http://gepia.cancer-pku.cn/index.html, accessed on 1 December 2021) [40] was used to confirm the differential expression found in the TIMER analysis by comparing the TCGA-KIRC (523 samples) with normal kidney samples from GTEx (Genotype-Tissue Expression project, http://www.gtexportal.org/home/index.html, accessed on 1 December 2021) (100 samples). The differential threshold of log2FC was 1 and the value cutoff of 0.05.

At the protein level, we used the UALCAN platform (http://ualcan.path.uab.edu/index.html, accessed on 1 December 2021) [41,42] to mine *GOT2* expression in the high-throughput mass spectrometry data, obtained from the Clinical Proteomic Tumor Analysis Consortium (CPTAC) of normal kidney tissues (*n* = 84) and primary KIRC tumors (N = 110) [43]. Integration and analysis of these data were described elsewhere [44,45]. Briefly, protein expression values (Log2 Spectral count ratio values) from CPTAC were first normalized within each sample profile, then normalized across samples. Then Z-values for each sample for GOT2 protein were calculated as standard deviations from the median across samples.

In this study, we checked the expression of GOT2 in the protein expression module of the HPA database (Human Protein Atlas, https://www.proteinatlas.org/, accessed on 1 December 2021) [46,47,48], and we analyzed the immunohistochemical results of GOT2 in tumor tissue (ID: 2176) and normal tissue (ID: 2067). The antibody used in both samples was HPA018139. All images of tissues stained by immunohistochemistry were manually annotated by a specialist, followed by verification by a second specialist. Protein expression score was based on immunohistochemical data manually scored concerning staining intensity (negative, weak, moderate, or strong) and the fraction of stained cells (<25%, 25–75% or >75%). Each combination of intensity and fractions was automatically converted into an protein expression level score as follows: negative—not detected; weak <25%—not detected; weak combined with either 25–75% or 75%—low; moderate <25%—low; moderate combined with either 25–75% or 75%—medium; strong <25%—medium, strong combined with either 25–75% or 75%—high (For more details, see https://www.proteinatlas.org/about/assays+annotation#ihk, accessed on 1 December 2021).

### 2.2. Clinical Correlations & Survival Analysis

Associations between clinicopathological parameters and mRNA expression of *GOT2* were analyzed using the UALCAN (http://ualcan.path.uab.edu/analysis.html, accessed on 5 December 2021) [41]. For these analyses, we included the following clinical features: cancer stage (stages 1, 2, 3, and 4), gender, tumor grade (1, 2, 3 and 4), KIRC subtypes (good risk: ccA; poor risk: ccB) [49], nodal metastasis status (N0: no regional lymph node metastasis; N1: metastases in 1 to 3 axillary lymph nodes).

The prognosis analysis was estimated by Kaplan–Meier (KM) survival curves generated by the Kaplan Meier (KM) Plotter (http://kmplot.com/analysis/, accessed on 5 December 2021) [50], GEPIA2 (http://gepia.cancer-pku.cn/index.html, accessed on 1 December 2021) [40] and HPA database (http://www.proteinatlas.org/, accessed on 5 December 2021) [46,47,48]. In this study, KIRC patients were split into high and low expression groups based on the median expression levels of *GOT2*, and then these two groups were compared in terms of relapse-free survival. Moreover, the hazard ratio (HR) with a 95% confidence interval (CI) and the *p*-value of the log-rank test were obtained. For all survival analyses, *p* < 0.05 was considered statistically significant.

### 2.3. GOT2 Methylation Analysis

To explore the DNA methylation level of all CpG islands located in GOT2 of KIRC-TCGA samples, we used the MethSurv database (https://biit.cs.ut.ee/methsurv/, accessed on 10 December 2021) [51]. Next, Shiny Methylation Analysis Resource Tool (SMART) (http://www.bioinfo-zs.com/smartapp/, accessed on 10 December 2021) [52] was used for differential methylation analysis of each *GOT2* probe and Spearman’s correlation between methylation level (β-values, 450 k array) and mRNA level (Log2-scaled, TPM+1). CpG-aggregated methylation values were determined by mean (β-values).

### 2.4. Analysis of Immune Cell Infiltration

We calculated and compared the *GOT2* gene expression contributed by different immune cell types in kidney samples (TCGA tumor/normal and GTEx normal) by the GEPIA2021 (http://gepia2021.cancer-pku.cn/, accessed on 1 December 2021) [53]. For each GTEx/KIRC-TCGA sample, we run the CIBERSORT algorithm (absolute mode) with the default parameters to obtain the absolute proportions of 22 immune cell subtypes. The 22 immune cells included: memory B cells, naïve B cells, activated memory CD4^+^ T cells, resting memory CD4^+^ T cells, naïve CD4^+^ T cells, CD8^+^ T cells, follicular helper T cells (Tfh), regulatory T cells (Tregs), and gamma/delta T cells, activated dendritic cells (DC), resting dendritic cells, eosinophils, macrophages (M0–M2), activated mast cells, resting mast cells, monocytes, resting NK cells, activated NK cells, neutrophils, and plasma cells. ANOVA (analysis of variance) was used for quantitative comparison. Sidak’s multiple comparisons test was used for the post-test, and *p* < 0.05 was considered significant.

### 2.5. Association between GOT2 and Tumor Microenvironment Exploration

The Tumor Immune Single-cell Hub (TISCH, http://tisch.comp-genomics.org/home/, accessed on 1 April 2022) is an online single-cell RNA-seq database focused on the tumor microenvironment (TME) [54]. In our analyses, two human KIRC scRNA-seq datasets [55,56] were used to obtain the *GOT2* average expression at the single-cell level. The expression of *GOT2* was collapsed by the mean value. The gene expression level displayed using UMAP and violin plots was quantified by the normalized values.

## 3. Results

### 3.1. Expression Level of GOT2 mRNA in Pan-Cancer

**Figure 1:**
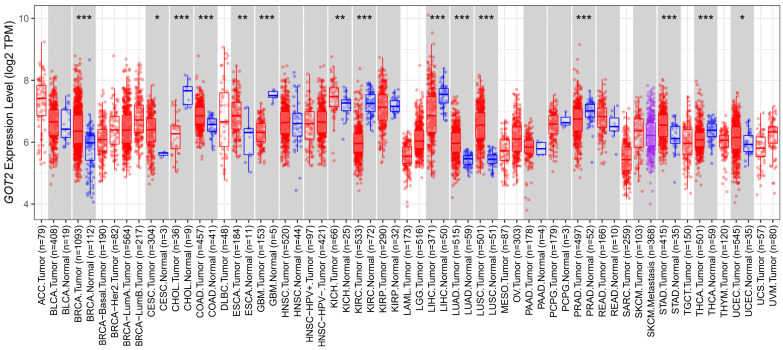
*GOT2* expression levels in pan-cancer (TCGA dataset). The box plot comparing specific *GOT2* expression in tumor samples (red plot) and paired normal tissues (blue plot) was derived from the TIMER database (* *p* < 0.05, ** *p* < 0.01, *** *p* < 0.001). TPM: transcripts per million.

To determine whether *GOT2* expression correlates with cancer, we surveyed *GOT2* expression in multiple cancer types and adjacent normal tissues through the TIMER database. As shown in Figure 1, compared with normal tissues, *GOT2* expression was higher in BLCA (Bladder Urothelial Carcinoma), CESC (Cervical squamous cell carcinoma and endocervical adenocarcinoma), COAD (Colon adenocarcinoma), KICH (Kidney Chromophobe), ESCA (Esophageal carcinoma), LUAD (Lung adenocarcinoma), LUSC (Lung squamous cell carcinoma), STAD (Stomach adenocarcinoma) and UCEC (Uterine Corpus Endometrial Carcinoma). Conversely, *GOT2* had markedly lower expression in CHOL (Cholangiocarcinoma), GBM (Glioblastoma), KIRC (Kidney renal clear cell carcinoma), LIHC (Liver hepatocellular carcinoma), PRAD (Prostate adenocarcinoma) and THCA (Thyroid carcinoma). All these data indicated that the dysregulation of this glutamic-oxaloacetic transaminase was common across several tumors, including KIRC.

### 3.2. GOT2 mRNA and Protein Are Downregulated and Correlated with Clinicopathological Parameters in KIRC

**Figure 2:**
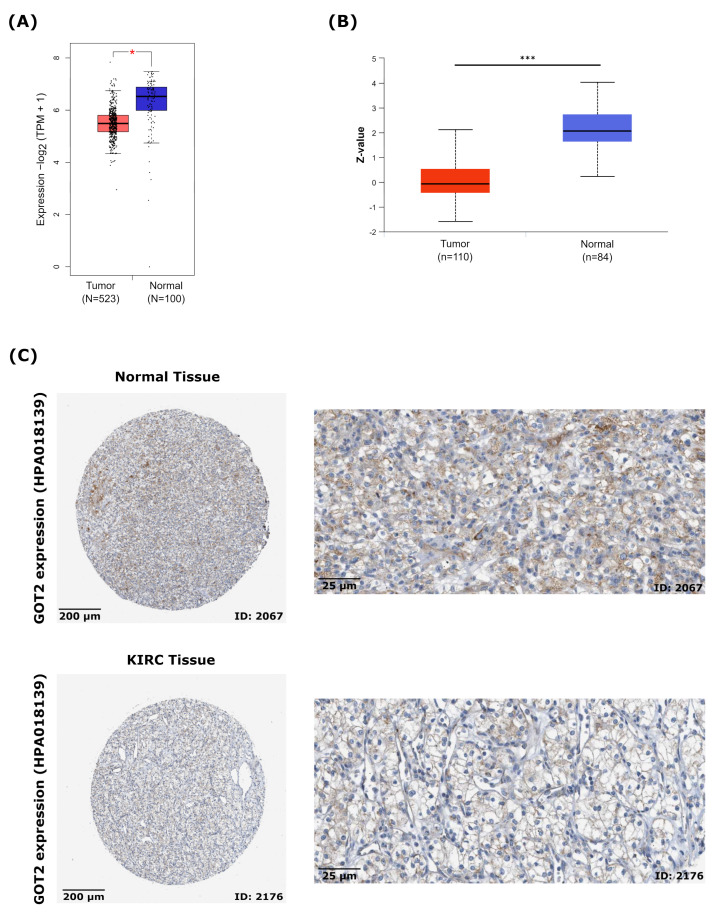
Expression of *GOT2* in KIRC and normal patients. (**A**) Differential expression of *GOT2* between KIRC samples from TCGA database (Red, *n* = 523 samples) and normal human kidney samples from GTEx database (Blue, *n* = 100 samples). (* *p* < 0.05, *** *p* < 0.001) (**B**) Significant downregulation of GOT2 protein level in the CPTAC KIRC cohort, analyzed by UALCAN. KIRC: *n* = 110; Normal samples: *n* = 84. Z-values represent standard deviations from the median across samples. (**C**) Representative images of immunohistochemical (IHC) staining of GOT2 protein in normal kidney tissue (Patient ID: 2067; Staining: medium; Intensity: moderate; Quantity: 75–25%; Location: cytoplasmic/membranous) and KIRC tissue (Patient ID: 2176; Staining: low; Intensity: weak; Quantity: >75%; Location: cytoplasmic/membranous) from the HPA database. Scale bars: left, 100 μm; right, 25 μm. Antibody used in both samples: HPA018139.

Next, to focus our analysis on KIRC, we investigated the transcription levels of *GOT2* performing a single-gene differential analysis using RNA-seq data from the TCGA database (KIRC-TCGA), compared with non-tumor tissues from the GTEx database by GEPIA2. Our results showed that the mRNA expression levels of *GOT2* in KIRC tissues (*n* = 523) were significantly lower than in adjacent normal tissues (*n* = 100) (Figure 2A). Correspondingly, in the CPTAC KIRC cohort, there was a significant downregulation of GOT2 in the tumors (Figure 2B), consistent with the immunohistochemical (IHC) staining images from the Human Protein Atlas (HPA) (Figure 2C). This further confirmed that the expression of *GOT2* in tumor tissues was significantly lower than that in normal tissues.

### 3.3. Relationship between GOT2 Expression and Clinical Pathological Parameters of Patients with KIRC

**Figure 3:**
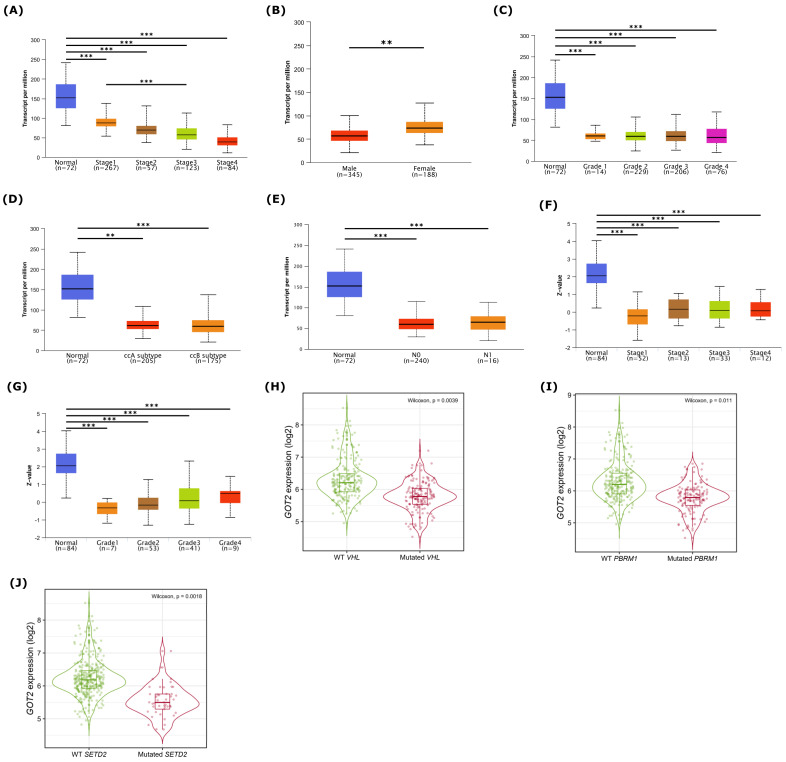
Association between *GOT2* expression and the clinicopathological features of KIRC patients. Box plots of *GOT2* mRNA expression according to: (**A**) KIRC stages (Stages 1, 2, 3 and 4). (**B**) gender (male, female). (**C**) KIRC grades (1, 2, 3 and 4). (**D**) clear cell renal cell carcinoma (ccRCC) good risk (ccA) and poor risk (ccB) subtype classification. (**E**) nodal metastasis status. GOT2 protein expression was differentially expressed in (**F**) clinical stages and (**G**) tumor grade ** *p* < 0.01, *** *p* < 0.001. *GOT2* expression in the KIRC cohort (TCGA) according to (**H**) VHL mutation status, (**I**) PBRM1 mutation status and (**J**) SETD2 mutation status, ** *p* < 0.01, *** *p* < 0.001.

We next investigated the correlation between clinical parameters and the *GOT2* expression in KIRC. Data showed that *GOT2* expression levels were significantly associated with stage, gender, grade, KIRC subtypes, and nodal metastasis status (Figure 3A–G). Lastly, concerning the most commonly mutated genes in KIRC, patients harboring *VHL*, *PBRM1*, and *SETD2* mutations under-expressed *GOT2* (Figure 3H–J). Thus, it is likely that *GOT2* expression may serve as a potential diagnostic biomarker for KIRC patients.

### 3.4. Low Expression of GOT2 Is Associated with Poor Outcome in KIRC Patients

**Figure 4:**
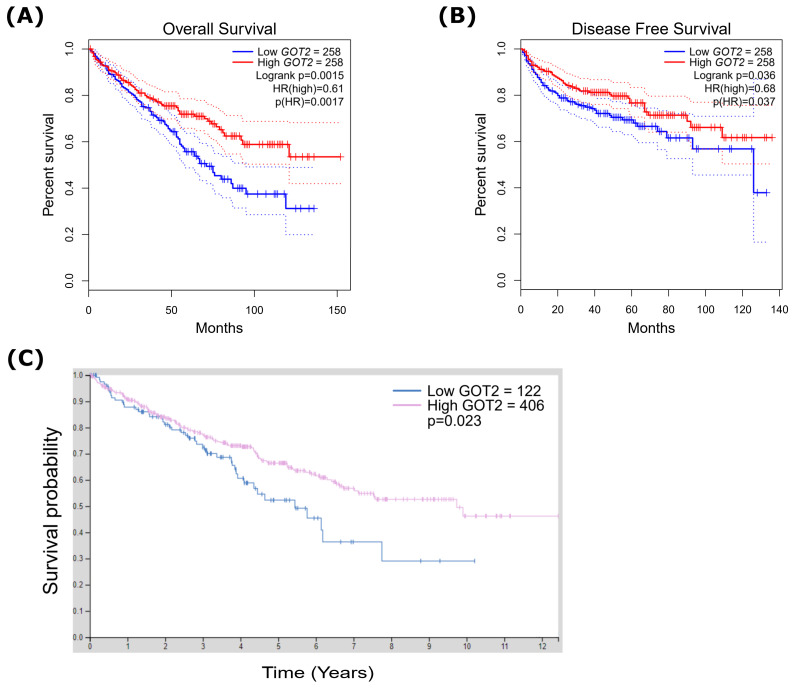
Kaplan–Meier survival analysis demonstrating the relationship between *GOT2* expression and prognosis in KIRC patients. Overexpression of *GOT2* mRNA prolonged (**A**) OS (*n* = 258) and (**B**) DFS (Disease-Free Survival; *n* = 258) of KIRC patients. (**C**) High expression of GOT2 protein prolonged OS of KIRC patients (*n* = 528) (*p* = 0.023). HR, hazard ratio; OS, overall survival; *GOT2*, Glutamic-Oxaloacetic Transaminase 2; KIRC, Kidney Renal Clear Cell Carcinoma.

Initially, to explore the influence of *GOT2* expression on KIRC outcomes, we conducted a Kaplan–Meier test and Cox regression analysis to delve into the associations with overall survival (OS) and disease-free survival (DFS). As shown in Figure 4A,B, the OS and DFS of KIRC patients with low expression of *GOT2* were significantly shorter than those with high expression. At the same time, we also noticed that the low level of GOT2 protein was significantly related to the worse OS (*p* = 0.023) (Figure 4C). Additionally, we investigated the relationship between *GOT2* expression and clinicopathological features of KIRC patients in the Kaplan–Meier plotter database. Surprisingly, low *GOT2* mRNA expression was correlated with worse OS in KIRC patients with stage 4 (HR = 0.56, *p* = 3.50 × 10^−2^), grade 3 (HR = 0.53, *p* = 7.90 × 10^−3^), and low mutation burden (HR = 2.28, *p* = 3.49 × 10^−2^) (Table 1). Here, the differences in the clinical characteristics suggest that the use of *GOT2* as an indicator gene should be carefully combined with the patient’s condition.

### 3.5. Hypermethylation of DNA in the Promoter Region Leads to Low Expression of GOT2 in KIRC

**Figure 5:**
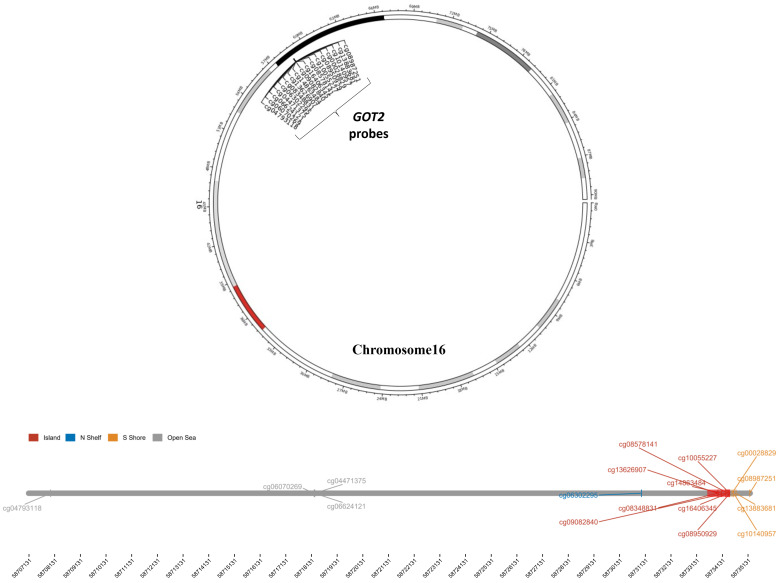
Chromosomal distribution of the methylation probes associated with *GOT2*. Upper panel: Circos plot depicting the genomic information of *GOT2* (16q21) and the probes used in this study. Lower panel: Segment plot showing the detailed information of genomic locations of each probe of *GOT2*, highlighting CpG island, N shelf, S Shore and Open Sea. The coverage of the promoter region is displayed as the red region (red box), which includes eight probes (cg08348831, cg13626907, cg14863484, cg09082840, cg16406345, cg08578141, cg10055227 and cg08950929). Numbers below represent the genomic length scale (1 kb).

**Figure 6:**
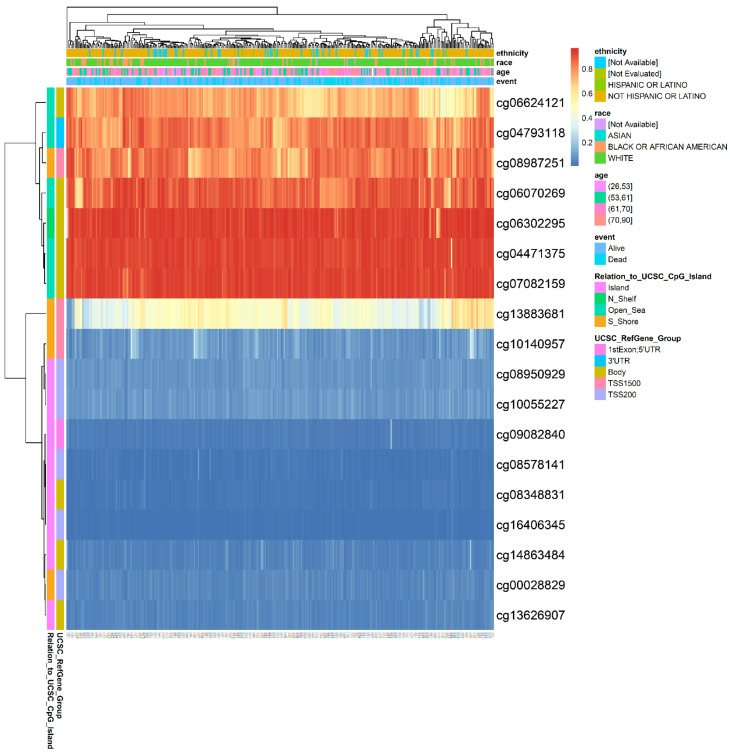
Dynamics of DNA methylation across all probes of *GOT2* in KIRC. Heat map showing the methylation levels of *GOT2* among different CpGs sites (probes) integrating ethnicity, race, age, vital status, and genomic regions of CpG sites (UCSC) from KIRC. Red to blue scale indicates high to low methylation levels.

**Figure 7:**
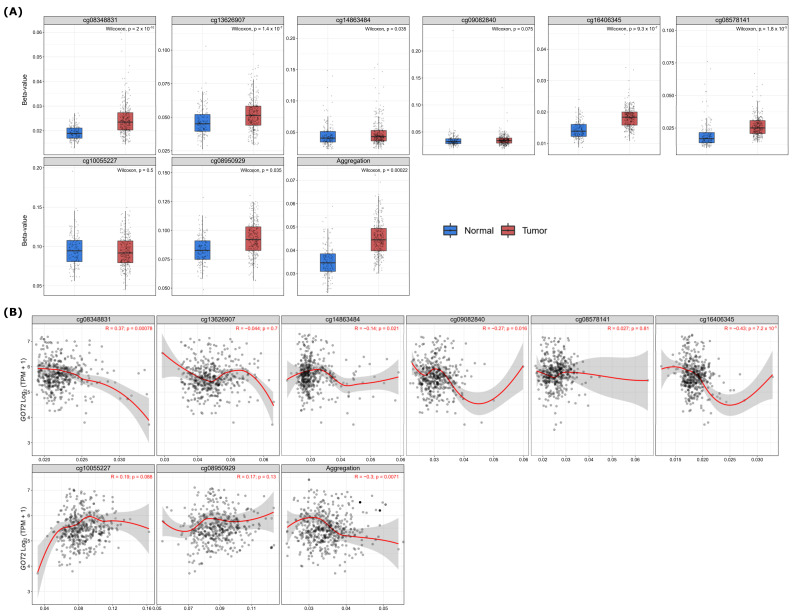
Hypermethylation of *GOT2* leads to downregulated expression in KIRC. (**A**) Differential methylation level of eighth *GOT2* probes (cg08348831, cg13626907, cg14863484, cg09082840, cg16406345, cg08578141, cg10055227 and cg08950929) between KIRC patients (*n* = 313) and normal samples (*n* = 157) from TCGA. (**B**) Spearman’s correlation between methylation level (β-values, 450 k array) and mRNA level (Log2-scaled, TPM + 1) of *GOT2* in KIRC samples from TCGA.

To further explore the epigenetic mechanism underlying *GOT2* underexpression, we analyzed the methylation level of seventeen probes covering the island (promoter region), N Shelf, S Shore, and Open Sea regions of *GOT2*, chosen through the UCSC Genome Browser (Table 2; Figure 5). Notably, the results showed that lower methylation levels for *GOT2* lay on probes at the promoter (island). At the same time, most hypermethylated sites fell in the open sea, N Shelf, and S Shore regions (Figure 6). Given that methylation of CpG sites within the gene promoter is a common mechanism in gene silencing, we next compared the methylation level of the probes that covered the *GOT2* promoter between normal vs. KIRC-TCGA samples (Figure 5). Interestingly, we found that the average methylation of all CpG sites (probes) near the TSS (transcription start site) of *GOT2* was significantly higher in tumor tissues than in the normal counterpart (Aggregation, *p* = 0.00022) (Figure 7A). Further analysis revealed a negative correlation between the methylation level and the mRNA of *GOT2* (Aggregation: R = −0.3, *p* = 0.0071) (Figure 7B), thus indicating that upregulation of DNA methylation level of CpGs island-associated promoter region may contribute to the downregulation of *GOT2* in KIRC patients.

### 3.6. GOT2 Expression Correlates with Immune Cell Infiltration in KIRC

**Figure 8:**
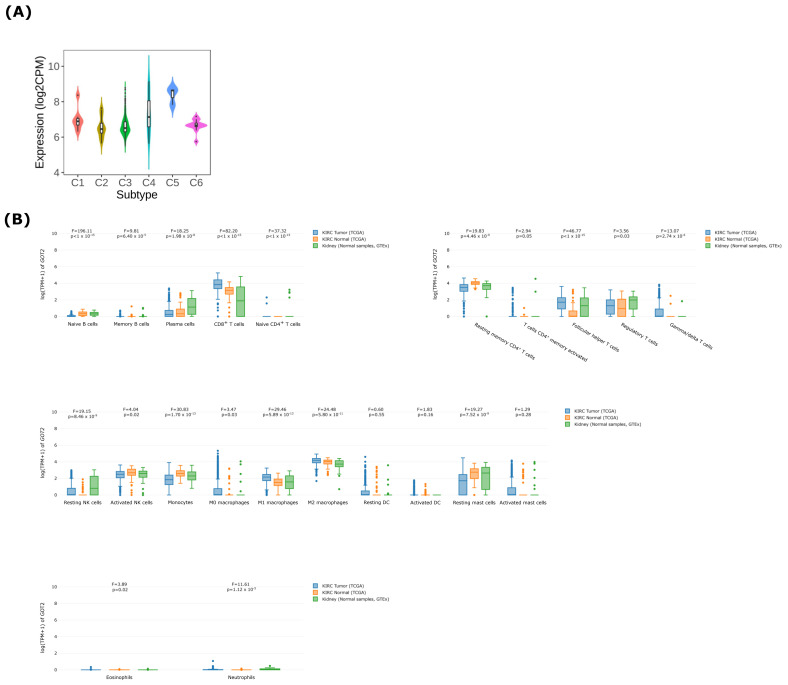
Association of *GOT2* expression, with immune subtypes and immune cell infiltration. (**A**) *GOT2* mRNA levels in TCGA-KIRC immune subtypes. C1: wound healing subtype (*n* = 7), C2: INF-γ dominant (*n* = 20), C3: inflammatory (*n* = 445), C4: lymphocyte depleted (*n* = 27), C5: immunologically quiet (*n* = 3), C6: TGF-ꞵ dominant (*n* = 16). One-way ANOVA *p*-value = 1.2 × 10^−4^. (**B**) *GOT2* expression in different immune cells types in KIRC samples from TCGA and normal samples from TCGA and GTEx.

Tumor-infiltrating immune cells are essential for immune response and prognosis in KIRC patients [57,58]. To determine whether *GOT2* could potentially impact immune cell infiltration in KIRC, we first examined the differences in *GOT2* expression across the six immune subtypes proposed by Thorsson et al. [59]. We observed that *GOT2* expression was highest in patients harboring the C5 subtype (immunologically quiet) and lowest in patients exhibiting the C2 subtype (IFN-gamma dominant), indicating that *GOT2* can be used as a marker for immunophenotyping of patients with clear-cell renal cell carcinoma (Figure 8A).

**Figure 9:**
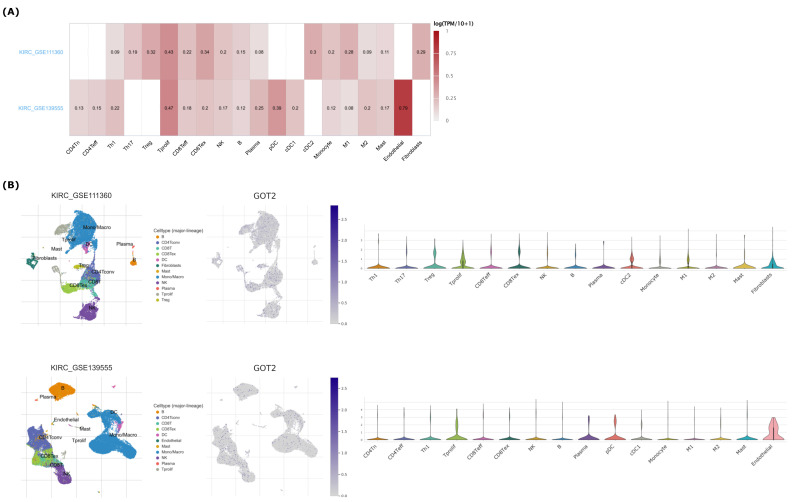
Expression of *GOT2* in scRNA-seq landscapes. (**A**) Heatmap of *GOT2* expression displayed heterogeneity in different clusters of cells in KIRC_GSE111360 [55] and KIRC_GSE139555 datasets [56]. (**B**) Expression of *GOT2* in GSE111360 (upper panel) and in GSE139555 (lower panel) datasets after Uniform Manifold Approximation and Projection (UMAP) processing. Violin diagrams depict the *GOT2* expression in different immune cells across each dataset analyzed.

**Figure 10:**
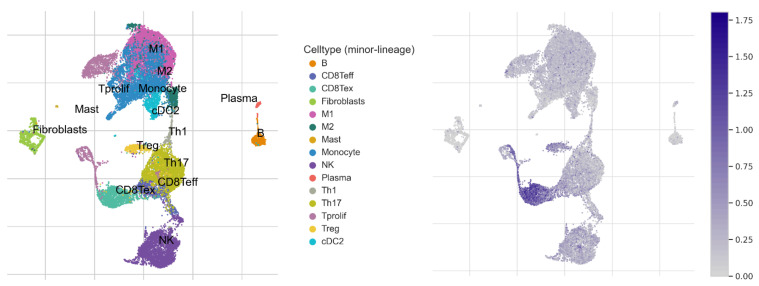
The expression source of the signature genes was revealed by single-cell analysis (GSE111360 dataset). The signature was composed of *GOT2*, *LAG3*, *CTLA4*, *EOMES*, *LGALS9*, *CD96*, *HAVCR2*, *PDCD1*, *TIGIT*, and *TOX*.

To better understand the role of *GOT2* in the infiltration of immune cells in KIRC, we used the CIBERSORT deconvolution analysis [53] for rough correlation analysis. The immune-related signatures revealed that *GOT2* was higher in CD8^+^ T cells, follicular helper CD4^+^ T (Tfh) cells, M1 and M2 Macrophages in KIRC-TCGA tumors than in normal tissues (Figure 8B). To further expand and strengthen these results, the analysis of two independent single-cell RNA sequencing (scRNA-seq) datasets [55,56] showed that *GOT2* was mainly expressed within endothelial cells, followed by proliferative T cells (Tprolif), plasmacytoid dendritic cells (pDCs), exhausted CD8^+^ T Cells (CD8Tex), Treg cells and conventional dendritic cells 2 (cDC2) (Figure 9A,B). These results imply that *GOT2* may play an essential role in the tumor microenvironment of the clear-cell renal cell carcinoma, affecting both stroma and immune cells. Interestingly, *GOT2* was broadly expressed within some clusters of immune cells (e.g., CD8ex and Tprolif) that also co-expressed some immune checkpoint inhibitors (e.g., *CTLA4*, *TIGIT*, *TOX*, *EOMES*, *LAG3*, *PDCD1*, *HAVCR2*, and *CD96*) (Figure 10), thus strongly suggesting that *GOT2* is involved in the dynamic regulation of immune homeostasis and is particularly relevant to T cell functionality.

## 4. Discussion

KIRC is characterized by profound metabolic reprogramming that involves multiple pathways [14,15]. Current evidence suggests that changes in the supply of specific metabolites, such as aspartate, which is essential for nucleotide and protein synthesis in proliferating cells and maintains the reducing potential [28,60,61], can function as opportunistic fuel sources for high proliferation and tumor growth [25,27,62]. As part of the malate–aspartate shuttle, mitochondrial *GOT2* generates aspartate from oxaloacetate and glutamate [63]. Additionally, this enzyme is involved in energy transduction, specifically amino acid metabolism and the urea and TCA cycles. Thus far, there is no available information about the detailed roles of *GOT2* in KIRC. Herein, we elucidated the most comprehensive insights into understanding the epigenetic regulation and the potential association of *GOT2* with the clinical and immunity of KIRC.

Based on a pan-cancer perspective, we initially demonstrated that *GOT2* is differentially expressed in 18 tumor types, thus potentially being a therapeutic target. Further interrogating KIRC, we showed that the *GOT2* mRNA and protein levels were markedly decreased in KIRC patients than in normal tissues. Besides, we observed that this transaminase was markedly lower as the pathological stage increased and was also strongly impacted by other clinicopathological characteristics, which conferred a worse outcome. Our findings are consistent with Zhao et al. [64], who also reported the decreased expression and prognostic value of *GOT2* in hepatocellular carcinoma (HCC). The results from a recent study support a scenario in which in *VHL*-deficient KIRC, but not in non-clear renal cell carcinomas (NCRCC), the simultaneous suppression of *GOT1* and *GOT2* is *HIF1*α-dependent, which impairs oxidative and reductive aspartate biogenesis [61]. Hence, to compensate for the low levels of aspartate seen in the KIRC [65], glutamine metabolism has a dominant role in sustaining KIRC growth [66]. This conceivably explains the down-expression of *GOT2* in KIRC patients harboring *VHL*, *PBRM1*, and *SETD2* mutations seen in our study, thus suggesting that *GOT2* repression represents a specific metabolic feature of KIRC.

DNA methylation of specific CpG sites in the promoter region is tightly linked with transcription repression. In the last few years, its role in carcinogenesis has been of considerable interest [67,68,69]. It is currently well known that KIRC is characterized by many epigenome aberrations [70,71]. Furthermore, many studies have pointed out the occurrence of a pattern known as CpG island methylator phenotype (CIMP) in 20% of KIRC [71,72]. However, no study has previously been carried out to analyze the role of DNA methylation in *GOT2* expression in KIRC. Here, for the first time, we provided evidence that the methylation of the *GOT2* promoter was increased in KIRC patients compared to normal samples. Additionally, the correlation analysis results revealed that promoter methylation was negatively correlated with the regulation of gene expression. According to these results, it can be speculated that the DNA hypermethylation in the promoter-associated CpG islands may be one of the mechanisms leading to *GOT2* down-expression in KIRC. However, additional efforts are necessary to determine the potential impact of additional events, such as chromatin structural modifications, miRNAs, and the influence of metabolites on patients exhibiting *GOT2* promoter hypermethylation.

In addition, another innovative aspect of this study clarified the significant correlations between *GOT2* expression and various tumor-infiltrating immune cells in KIRC. Previous studies have found that T cells and macrophages represented the dominant populations in most KIRC cases [73,74], consistent with our findings, which indicates that *GOT2* expression was more likely to affect the tumor infiltration of subtypes of T cells, especially CD8^+^ T cells and follicular helper CD4^+^ T (Tfh) cells, and M1 and M2 macrophages compared to normal renal tissue. Our deep-dissection of individual cell subsets from scRNA-seq data revealed that *GOT2* was broadly expressed within exhausted CD8^+^ T Cells (CD8Tex) and in the proliferative T cells (Tprolif). Unlike all solid tumors, high tumor-infiltrating CD8^+^ T-cells predicted poor overall survival and inferior therapeutic responsiveness in patients with KIRC [75,76,77]. However, a comprehensive characterization of immune cells from KIRC patients using scRNA-seq along with T-cell-receptor (TCR) sequencing revealed that CD8^+^ T-cells exhibited four distinct groups that may represent transcriptional states upon tumor infiltration with distinct prognostic significance: two of them were associated with a *PD-1*^+^ *TIM-3*^+^ exhausted subcluster, one with a proliferative subcluster, and a fourth with the higher levels of cytokine signaling [73]. Moreover, a correlation observed between increased clusters with the signature CD8_6 (CD8^+^T-cells) and TAM_3 (macrophages) showed a better prognosis. In another study, a first-in-class CAR T-cell therapy co-expressing GOT2 enhanced T-cell metabolic function for treating GPC3-positive solid tumors, supporting the progress of a future first-in-human trial in subjects with GPC3-positive tumors [78]. Considering this context, we argue that *GOT2* is likely to play distinct roles at different stages of T-cell exhaustion and might potentially be modulated by the spectrum of changes in TME conditions of KIRC patients, including tumor metabolism, hypoxia, nutrient restriction, and exhaustion driven by chronic stimulation, thus strengthening the potential application of synergic modulation of the *GOT2* and T cell exhaustion markers in non-responsive KIRC patients to boost antitumor and immune responses.

In conclusion, using a series of rigorous bioinformatics analyses, we showed that the mRNA and the expression levels of the *GOT2* protein were significantly decreased in KIRC patients compared to normal ones. This low expression was positively associated with clinicopathological features, culminating in poor clinical outcomes for KIRC patients. Notably, we provide the first mechanism insights into the epigenetic-mediated regulation of *GOT2*, which is driven by the DNA methylation in the promoter-related CpG islands. Finally, we also shed light on the influence of *GOT2* expression in immune cell infiltration, suggesting that *GOT2* may be a potential prognostic marker and therapeutic target for KIRC patients.

## Figures and Tables

**Table 1:** Correlation of *GOT2* mRNA expression and clinical outcomes in KIRC from TCGA database.

Clinicopathological Characteristics	*n*	Hazard Ratio (95% CI)	Logrank *p*
**Stage**			
1	265	1.67 (0.92–3.03)	8.94 × 10^−2^
2	57	0.29 (0.06–1.31)	8.56 × 10^−2^
3	123	0.35 (0.74–2.39)	3.47 × 10^−1^
4	82	0.56 (0.32–0.97)	**3.50** **× 10^−2^**
**Gender**			
Female	186	0.62 (0.36–1.05)	7.45 × 10^−2^
Male	344	0.71 (0.47–1.06)	9.56 × 10^−2^
**Grade**			
1	14	-	-
2	227	1.51 (0.8–2.84)	2.04 × 10^−1^
3	206	0.53 (0.33–0.85)	**7.90 ×10^−3^**
4	75	0.64 (0.35–1.19)	1.60 × 10^−1^
**Mutation burden**			
High	168	1.34 (0.77–2.34)	2.94 × 10^−1^
Low	164	2.28 (1.04–4.99)	**3.49** **× 10^−2^**
**Race**			
White	459	0.72 (0.5–1.02)	6.17× 10^−2^
Asian	8	-	-
Black/African American	56	0.39 (0.12–1.29)	1.10 × 10^−1^
**Hemoglobin result**			
Elevated	5	1.73 (0.1076–27.8905)	6.98 × 10^−1^
Normal	184	0.70 (0.3784–1.3129)	2.70 × 10^−1^
Low	261	1.22 (0.8484–1.7628)	2.81 × 10^−1^
**Laterality**			
Right	280	1.12 (0.7181–1.7561)	6.11 × 10^−1^
Left	248	0.71 (0.4721–1.0682)	1.00 × 10^−1^
Bilateral	4	-	-
**Serum calcium result**			
Elevated	10	0.65 (0.1599–2.6446)	5.48 × 10^−1^
Low	203	0.79 (0.5749–1.5257)	7.92 × 10^−1^
Normal	150	0.62 (0.376–1.0547)	7.88 × 10^−2^

Bold numbers indicate a statistically significant correlation with a *p*-value less than 0.05. Abbreviations: CI = confidence interval.

**Table 2:** List of the 17 probes analyzed. CGI: CpG islands.

Probe	Chromosome	Start	End	CGI Position
cg04793118	chr16	58707974	58707975	Open Sea
cg06070269	chr16	58718235	58718236	Open Sea
cg06624121	chr16	58718247	58718248	Open Sea
cg04471375	chr16	58718261	58718262	Open Sea
cg06302295	chr16	58730982	58730983	N Shelf
cg08348831	chr16	58733959	58733960	Island
cg13626907	chr16	58733963	58733964	Island
cg14863484	chr16	58734106	58734107	Island
cg09082840	chr16	58734264	58734265	Island
cg16406345	chr16	58734350	58734351	Island
cg08578141	chr16	58734361	58734362	Island
cg10055227	chr16	58734416	58734417	Island
cg08950929	chr16	58734423	58734424	Island
cg00028829	chr16	58734507	58734508	S Shore
cg10140957	chr16	58734573	58734574	S Shore
cg13883681	chr16	58734661	58734662	S Shore
cg08987251	chr16	58735200	58735201	S Shore

## Data Availability

The datasets analyzed for this study can be found in the GEPIA2 dababase (http://gepia.cancer-pku.cn/index.html, accessed on 1 December 2021), GTEx (http://www.gtexportal.org/home/index.html, accessed on 1 December 2021), HPA database (Human Protein Atlas) (https://www.proteinatlas.org/, accessed on 1 December 2021), UALCAN (http://ualcan.path.uab.edu/analysis.html, accessed on 1 December 2021), Kaplan Meier (KM) Plotter (http://kmplot.com/analysis/, accessed on 5 December 2021), MethSurv database (https://biit.cs.ut.ee/methsurv/, accessed on 10 December 2021), Shiny Methylation Analysis Resource Tool (SMART) (http://www.bioinfo-zs.com/smartapp/, accessed on 10 December 2021), GEPIA2021 (http://gepia2021.cancer-pku.cn/, accessed on 1 December 2021) and Tumor Immune Estimation Resource (TIMER, https://cistrome.shinyapps.io/timer, accessed on 1 December 2021 ), Tumor Immune Single-cell Hub (TISCH, http://tisch.comp-genomics.org/home/, accessed on 1 April 2022). Further inquiries can be directed to the corresponding authors.
